# The impact of the global budget payment on healthcare fund expenditure in integrated delivery systems: evidence from China’s county-level medical alliance reform

**DOI:** 10.3389/fpubh.2026.1837561

**Published:** 2026-07-31

**Authors:** Kan Wu, XiuLi Wang

**Affiliations:** 1 Department of Epidemiology and Health Statistics, West China School of Public Health and West China Fourth Hospital, Sichuan University, Chengdu, Sichuan, China; 2 Institute for Healthy Cities and West China Research Center for Rural Health Development, Sichuan University, Chengdu, Sichuan, China

**Keywords:** county-level healthcare consortium, global budget payment, health insurance fund expenditure, integrated healthcare delivery system, machine learning

## Abstract

**Background:**

The global budget payment is a key policy tool for incentivizing integrated healthcare delivery systems to pursue financial sustainability. However, there is little empirical evidence showing its effects. This study evaluates the impact of the health insurance global budget payment reform implemented under China’s county-level integrated healthcare consortium model on the efficiency of health insurance fund expenditure. The study also examines the heterogeneous effects across different service types.

**Methods:**

The study used panel data from L County, and constructed a quasi-natural experiment framework. The dependent variables: total pooled fund expenditure, general outpatient fund expenditure, outpatient fund expenditure for chronic/special diseases, and inpatient fund expenditure. The independent variable was a policy dummy variable representing the implementation of the consortium-based global budget payment reform. A two-way fixed effects model controlled for unobserved county-level heterogeneity and time trends. Potential endogeneity and model robustness were assessed using a random effects model with the Hausman-Taylor estimator and a series of robustness checks.

**Results:**

Baseline regressions indicate that policy implementation is significantly negatively correlated with total medical insurance expenditures (H1) and inpatient expenditures (H4) (*p* < 0.01). No significant effect is seen for outpatient (H2) or chronic and special disease expenditures (H3). The fixed-effects model further confirms the negative effects on H1 and H4 (*p* < 0.01) and shows a significant positive association with outpatient expenditures (*p* < 0.05). This indicates potential cost-shifting effects. Both the Breusch–Pagan test (*p* < 0.05) and the Hausman test support the adoption of the fixed-effects model. The results remain robust in the two-way fixed-effects specification. Model explanatory power varies: total and inpatient expenditure models show relatively high explanatory capacity (*R*^2^ = 0.273–0.538), while the chronic and special disease model shows a weak fit (*R*^2^ = 0.031).

**Conclusion:**

Within China’s county-level integrated healthcare consortium framework, the health insurance global budget payment reform effectively curbs the overutilization and growth in costs of inpatient services, but may induce a shift in services towards outpatient care. This cost-shifting effect indicates that isolated inpatient global budgeting, without a coordinated payment mechanism design for outpatient services, may not optimize the overall expenditure efficiency of health insurance funds.

## 1 Introduction

Globally, the continued rise in healthcare expenditure is a significant challenge for national fiscal and health insurance systems. Reforming payment methods from traditional fee-for-service models towards value-based prospective payment systems (where providers receive a fixed amount based on patient outcomes, quality, and efficiency) has become a central topic in health economics and policy (1). The global budget payment stands out as a forward-looking model. It sets a fixed total payment for a defined enrolled population or healthcare delivery system over a specific period (typically 1 year) (2). The goal is to fundamentally change the provider’s incentive from a revenue-centric orientation. The approach compels providers to optimize resource allocation and increase service efficiency within the budgetary cap, imposing a hard constraint on healthcare cost growth. Within integrated healthcare delivery systems [e.g., Accountable Care Organizations in the United States (U.S.) and Integrated Care Systems in the United Kingdom (UK)], global budgets are often combined with mechanisms like capitation and shared savings (3). This approach is an effective policy tool for coordinating care across different levels of institutions, promoting preventive care, and reducing unnecessary hospitalizations and specialist service use (4).

There is substantial literature on the impact of global budgeting on healthcare fund expenditures within integrated delivery systems. Consensus has been reached on some topics, while there is ongoing debate for others (5). International evidence consistently demonstrates that in integrated systems, such as Health Maintenance Organizations (HMOs), global budgeting effectively curbs excessive growth in medical expenditures (6). It does this by imposing strong budgetary constraints and sharing financial risk. The core mechanism lies in shifting the payment paradigm from a traditional, fee-for-service, retrospective model to a prospective, value-based model that holds providers accountable for health outcomes. This incentivizes providers to proactively control costs and reduce unnecessary services (7). However, the research highlights potential risks with this approach. First, it may induce the under-provision of care or risk-driven selection, where providers avoid high-cost patients or reduce necessary services to control spending. Second, it may lead to cost-shifting, such as transferring costs from inpatient to outpatient settings or from within-hospital to out-of-hospital services. This can result in unintended changes in the expenditure structure of the funds, rather than achieving fundamental savings in the total amount (8).

In the Chinese context, particularly the integrated reforms represented by county-level medical alliances, research is emerging but needs to be further deepened. Most studies acknowledge the preliminary effectiveness of global budgeting in slowing the growth rate of total medical insurance fund expenditures within Yigongti. This confirms its role as a “steering mechanism” to guide resource allocation towards primary care and disease prevention. However, evidence largely comes from macro-aggregate analyses or descriptive case studies (9). More rigorous, empirical testing, based on large-sample panel data, is needed to examine the micro-level mechanisms through which global budgeting specifically affects the internal expenditure structure of the funds. This includes evaluating trade-offs between spending on general outpatient care, chronic/special disease outpatient care, and inpatient care (10). Further, previous studies do not fully account for key control variables in the reform context (e.g., enrollee mix, institutional type heterogeneity). This makes it difficult to precisely isolate the net policy effect. This context highlights the need to thoroughly investigate the structural impact and transmission pathways of global budgeting on medical insurance fund expenditures within the specific organizational form of Yigongti. This would address outstanding theoretical debates and provide a precise evidence base for optimizing payment system reforms with Chinese characteristics (9, 10).

To address these research gaps, this study creates a quasi-natural experiment using the pilot reform in L County of a Chinese province to construct a rigorous research framework. The study evaluates the effect of the healthcare insurance fund global budget payment reform on county-level insurance fund expenditure (11). Specifically, this study addresses three core research questions. (1) Does the global budget payment reform effectively control the growth of total healthcare insurance fund expenditure? (2) What heterogeneous impacts does this reform have on fund expenditures for different service types (inpatient, general outpatient, and outpatient chronic/special disease care), and what provider behavioral changes do these reflect? (3) Does the reform effect show significant heterogeneity across different healthcare institutions and over time? By situating the internationally recognized theory of global budget payment within the specific context of China’s healthcare consortiums, this research adds to theoretical descriptions of the mechanisms through which payment reforms operate within tiered healthcare systems (12). The study applies rigorous econometric models, complemented by robustness checks, to provide reliable causal inferences and evidence for policy evaluation (13). Further, the study describes the profound impact of the reform on the expenditure structure of the healthcare insurance fund. This leads to precise and actionable empirical insights for policymakers to optimize payment scheme design. This provides significant reference value for deepening payment method reforms in China and other countries with similar contexts (10, 13).

## 2 Policy context

China’s healthcare system is at a critical juncture, transitioning from scale expansion to a focus on quality and efficiency. To establish a health-centered service system, the Chinese government has promoted the development of County-level Healthcare Consortiums since 2017. These integrate county-level hospitals, township health centers, and village clinics within a county into a single community of shared responsibility, management, service, and interests. Each consortium has a goal of establishing a tiered diagnosis and treatment system; this conflicts with the traditional fee-for-service payment model (3, 11). Fee-for-service incentivizes service volume over health outcomes, leading to resource concentrations in high-profit inpatient services and advanced diagnostic procedures (4, 13). China’s National Healthcare Security Administration launched a pilot reform in 2019: implementing a global budget payment for consortium-level healthcare insurance funds. The core approach is to prepay the total healthcare insurance fund to the entire consortium on a per capita basis, coupled with an incentive mechanism to retain savings and share reasonable overruns (5). The goal is to transform the consortium from a passive network of service providers into a quasi-Health Maintenance Organization that proactively assumes responsibility for health outcomes and financial performance for its enrolled population (6).

In November 2017, L County established a closely-knit county-level healthcare consortium. This consortium was anchored by a county-level people’s hospital operating at tertiary management standards and integrated all healthcare institutions within the county, including two secondary hospitals, seven township health centers, one community health service center, and dozens of village clinics. This formed a unified, tiered diagnosis and treatment network. In 2022, the county was designated as a national-level pilot site, deepening the transition of its integrated model. This marked the start of the policy implementation period analyzed in this paper. The consortium’s core objectives were to synergize resources, strengthen primary care service capacity, and assume responsibility for the health outcomes of approximately 240,000 insured residents within its jurisdiction.

Prior to the reform, L County operated under a traditional fee-for-service payment model. A lack of cost constraints led to rapid growth in healthcare costs and increased pressure on healthcare insurance fund expenditures. The reform started in 2022, with the initial implementation of a global budget for inpatient expenses at the major county-level hospital. This marked the beginning of the payer (the healthcare insurance fund) transferring financial risk to the provider (the hospital). Within the overarching framework of the global budget, L County refined its management by progressively introducing multiple payment tools. The next key policy change involved shifting the payment unit from individual hospitals to the entire consortium. The healthcare insurance authority implemented a consortium-level payment mechanism featuring global prepayment, savings retention, and overrun sharing. Specifically, inpatient services were settled internally using a points-based system under the Diagnosis-Intervention Packet (DIP) framework. Outpatient services adopted a capitation payment model. This change aligned the economic incentives of all institutions within the consortium. The goal was to incentivize the system to proactively control costs, improve efficiency, and focus on health management and disease prevention.

## 3 Data and methods

### 3.1 Research data

The empirical analysis of this study was conducted using data from the medical fund settlement system of L County. During the data preprocessing stage, records related to non-county medical alliances, such as private medical institutions and clinics, were excluded. Entries with missing key variables or logical inconsistencies were removed. This resulted in a final sample of 3,262,817 valid reimbursement records. The data cover 10 medical institutions spanning the period from September 2021 to December 2024, which covers both pre-and post-policy implementation periods. To align with the research design, the raw data were aggregated by month to construct a panel dataset with medical institutions as the observational units. The reform measures are gradually implemented on a monthly basis (such as monthly budget settlements), and weekly data would introduce too much noise (such as weekend effects and holiday disruptions).

**Figure 1:**
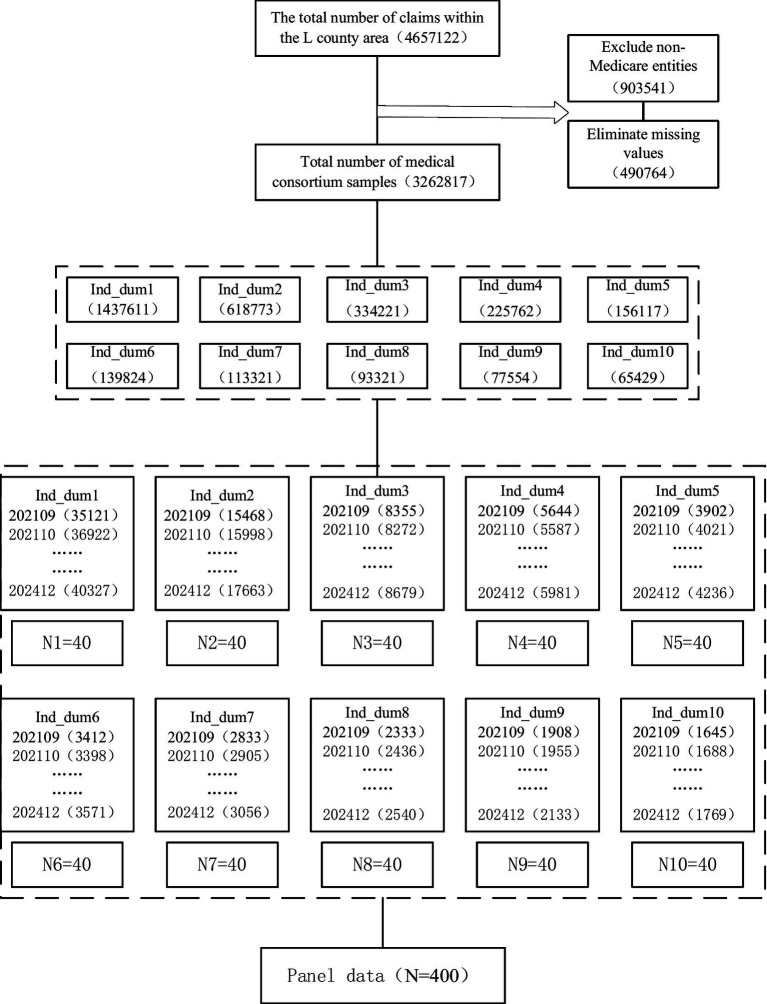
Data screening and sample selection process for panel data analysis.

The core variables encompass two dimensions: (1) medical institution characteristics, including a unique institution identifier and institution type (e.g., county-level hospital, township health center); and (2) patient and cost characteristics, comprising patient demographic information (age, sex), visit date, type of medical insurance enrollment, and categorized fund expenditure variables (e.g., pooled fund expenditure, outpatient expenditure, inpatient expenditure). For the expenditure variables, monthly means and standard deviations were further calculated for each medical institution. Following this processing, a balanced panel dataset was obtained with an “institution-month” observation structure, consisting of 400 observations across 10 medical institutions over 40 months. Details are provided in Figure 1.

### 3.2 Research variables

The following variables were selected to investigate the mechanism by which the reformed global budget payment method impacts the county medical consortium. (1) Dependent Variables: Indicators related to healthcare fund expenditure, including the total pooled fund expenditure, general outpatient fund expenditure, outpatient fund expenditure for chronic/special diseases, and inpatient fund expenditure. These measure changes over time in the efficiency of healthcare fund utilization. (2) Core Independent Variable: Policy variable indicating the presence of the reform of the global budget payment method for healthcare funds within the county medical alliance. (3) Control Variables: Based on a literature review, control variables include year, age, gender, health insurance type, and institution type. The specific variable names and their coding are detailed in Table 1.

**Table 1:** Variable definitions and descriptions.

Variable category	Variable name	Abbreviation	Definition and measurement
Dependent variables	Total medical insurance fund expenditure ( Y₁ _1_)	Expense_Total	The actual expenditure amount from the medical insurance fund per medical encounter, covering both outpatient and inpatient services
	Outpatient medical insurance fund expenditure ( Y₂ _2_)	Expense_OUT	The average expenditure amount from the medical insurance fund per outpatient visit
	Fund expenditure for chronic and special diseases ( Y₃ _3_)	Expense_CSD	The average expenditure amount from the medical insurance fund per outpatient visit specifically for chronic and special diseases
	Inpatient medical insurance fund expenditure ( Y₄ _4_)	Expense_INP	The average expenditure amount from the medical insurance fund per inpatient admission
Independent variable	Payment reform dummy ( D )	Post	Indicates the phase of the county-level medical alliance payment reform:
			0 = Pre-reform period (Sep 2021–May 2022, Control Group);
			1 = Post-reform period (Jun 2022–Dec 2024, Treatment Group)
Control variables	Gender ( $X_{1})$	Gender	Patient’s gender:
			0 = Female;
			1 = Male
	Age ( $X_{2})$	Age	Patient’s age at the time of the medical encounter (unit: years)
	Type of medical insurance ( $X_{3})$	INS_type	Type of health insurance the patient is enrolled in:
			0 = Urban and Rural Resident Basic Medical Insurance;
			1 = Urban Employee Basic Medical Insurance
	Hospital type ( $X_{4})$	HOS_type	Administrative level of the healthcare institution where the encounter occurred:
			1 = Secondary and above hospitals;
			2 = Primary hospitals
	Year ( $X_{5})$	Medi_year	Year in which the medical encounter occurred:
			1 = 2022;
			2 = 2023;
			3 = 2024

### 3.3 Research hypotheses

Table 2 lists the core hypotheses of the study, which focus on two dimensions. The first dimension focuses on the changes in the efficiency of medical insurance fund expenditures. We hypothesize that implementing the reform of the tight county medical consortium has a significant controlling effect on the total expenditure of medical insurance funds (H1) and its main components: outpatient fund expenditure (H2), chronic and special disease fund expenditure (H3), and inpatient fund expenditure (H4). The second hypothetical dimension involves the heterogeneity of effects. Hypotheses H5 and H6 propose that the model has significant individual (institutional-level) fixed effects and time fixed effects, respectively. These unobserved heterogeneities may significantly impact the expenditure results.

**Table 2:** Research hypothesis.

Dimension	Research hypothesis	Hypothesis content
Changes in fund expenditure efficiency	H1	Following the implementation of the county medical alliance reform, the overall expenditure of the medical insurance fund has significantly decreased, indicating that the reform has a cost-control effect on the total fund expenditure
	H2	The county medical alliance reform contributes to curbing the growth of fund expenditure in outpatient services, with the total outpatient fund expenditure showing a significant downward trend after the reform
	H3	Post-reform, the fund expenditure related to outpatient services for chronic and special diseases has been effectively controlled, reflecting the cost-containment effectiveness of the medical alliance in managing chronic and special diseases
	H4	Under the integrated management model of the medical alliance, the growth rate of fund expenditure for inpatient services has slowed down or declined, suggesting that the reform imposes a certain restraining effect on hospitalization costs
Heterogeneity analysis	H5	There are significant individual fixed effects among different medical institutions, indicating that institution-level heterogeneity factors have an important impact on medical insurance fund expenditure
	H6	Changes in medical insurance fund expenditure are significantly influenced by time fixed effects, reflecting that the policy effects exhibit dynamic evolution characteristics over time

### 3.4 Research methods

To effectively control for unobservable individual heterogeneity and test the robustness of the estimation results, this study constructs and analyzes a balanced panel dataset from L County spanning 2021–2024. The following econometric model is constructed for empirical analysis. Specifically, the Pooled Ordinary Least Squares (POLS) model, the Fixed Effects (FE) model, and the Random Effects (RE) model are used for estimation. Comparing model results and using the Hausman test to select between the fixed and random effects models, maximizes the reliability of the core findings. The specific specifications of each model are discussed in the following subsections.

#### 3.4.1 Pooled ordinary least squares (POLS)

The Pooled OLS method is a statistical approach for panel data analysis that treats the panel data as a large pooled cross-sectional dataset. It does not consider individual and time heterogeneity and uses Ordinary Least Squares regression. The specific model is specified as follows Equation 1:


$$\begin{array}{l} EP_{it}=\alpha +\beta_{1}POLS_{it}+\beta_{2}Age_{it}+\beta_{2}{\mathrm{Gender}}_{it}\\ +\beta_{3}Insurance_{it}+\beta_{4}Hospital_{it}+\varepsilon_{it} \end{array}$$ (1)

where $EP_{it}$ represents the observed outcome for the total medical insurance fund, outpatient, chronic/special disease, and inpatient fund expenditures for the i-th cross-sectional unit at time α . This is the intercept common to all individuals. The term $POLS_{it}$ is the core independent variable, indicating the status before and after implementing the global budget policy. The term β is a k×1 vector of estimated coefficients, with βi representing the regression coefficient for each variable. The term $\varepsilon_{it}$ is the independently and identically distributed error term.

#### 3.4.2 Fixed effects (FE) and random effects (RE) models

Both fixed and random effects models are designed to control for omitted time-invariant individual characteristics. Control variables only include features that change over time at the institutional level, while year effects are treated as fixed effects for non-time-varying shocks. This control is achieved either by demeaning the data within each individual (within the transformation) or by including individual dummy variables. These characteristics are correlated with the individual and do not change over time; as such, these methods can effectively account for them. The general forms of the models are as follows. Equation 2 represents the Fixed Effects (FE) model, and Equation 3 represents the Random Effects (RE) model.


$$\begin{array}{l} EP_{it}=\alpha +\beta_{1}FE_{it}+\beta_{2}Age_{it}+\beta_{2}{\mathrm{Gender}}_{it}\\ +\beta_{3}Insurance_{it}+\beta_{4}Hospital_{it}+\gamma_{i}+\left(\varepsilon_{it}\right) \end{array}$$ (2)

$$\begin{array}{l} EP_{it}=\alpha +\beta_{1}RE_{it}+\beta_{2}Age_{it}+\beta_{2}{\mathrm{Gender}}_{it}\\ +\beta_{3}Insurance_{it}+\beta_{4}Hospital_{it}+\left(\gamma_{i}+\varepsilon_{it}\right) \end{array}$$ (3)

where i denotes different individuals, and t denotes different years. The terms *α* and *β* are the conventional intercept and slope parameters, respectively. In the FE model, the omitted individual characteristic $\gamma_{i}$ is treated as a variable that varies across individuals i but not over time t. It is incorporated as part of the explanatory variables (via dummy variables or transformation). In the RE model, $\gamma_{i}$ is treated as a component of the composite error term uit, which is assumed to be uncorrelated with the explanatory variables.

#### 3.4.3 Breusch–Pagan test

The Breusch–Pagan (BP) test examines whether the variance of the random error term relates to the explanatory variables. Specifically, it tests for a linear relationship between the squared errors (or their variance) and the explanatory variables. A significant relationship indicates there is heteroskedasticity.

The procedure is as follows Equation 4:

① Estimate the primary regression model:


$$Y_{i}=\beta_{0}+\beta_{1}x_{i1}+\beta_{2}x_{i2}+\ldots +\beta_{n}x_{in}+\mu_{i}$$ (4)

where $Y_{i}$ is the dependent variable; $x_{i1}\ldots \ldots x_{in}$ are the independent variables; and $\mu_{i}$ is the error term.

② Calculate the test statistic:

Estimate the primary regression model using OLS to obtain the residuals $e_{i}=Y_{i}-{\hat{Y}}_{i}$ . Then, run an auxiliary regression of the squared residuals $e_{i}^{2}$ on the independent variables Equation 5:


$$e_{i}^{2}=\delta_{0}+\delta_{1}x_{i1}+\delta_{2}x_{i2}+\ldots +\delta_{n}x_{in}+\varepsilon_{i}$$ (5)

where $\delta_{i1}\ldots \ldots x\delta_{in}$ are parameters to be estimated, and $\varepsilon_{i}$ is a new error term. The next step is to compute the coefficient of determination $\;R^{2}$ from this auxiliary regression. The BP test statistic is calculated as $\;BP=n\times R^{2}$ , where n is the sample size.

③ Conduct hypothesis testing:

The Null Hypothesis $H_{0}$ : $\delta_{1}=\delta_{2}=\ldots =\delta_{n}=0$ (Homoskedasticity).

The Alternative Hypothesis $H_{1}$ : $\delta_{1}=\delta_{2}=\ldots =\delta_{n}\neq 0$ (Heteroskedasticity).

Under the null hypothesis and in large samples, the BP statistic asymptotically follows a chi-squared distribution with a number of degrees of freedom that are equal to the number of independent variables k. If the calculated BP value exceeds the critical chi-squared value (or if the corresponding *p*-value is less than the chosen significance level), the null hypothesis is rejected. This indicates there is heteroskedasticity.

#### 3.4.4 Hausman test

The Hausman test states that if two estimators are both consistent, their estimates should not differ systematically. A significant difference between the estimates indicates that one of the estimators is inconsistent. This may be due to a correlation between the individual effects and the regressors in the RE model.

The procedure is as follows:

① Estimate both models and compute the statistic:

Estimate the parameters using both the Fixed Effects ( ${\hat{\beta }}_{FE}$ ) and Random Effects ( ${\hat{\beta }}_{RE}$ ) models. The Hausman test statistic is calculated as Equation 6:


$$H={\left({\hat{\beta }}_{FE}-{\hat{\beta }}_{RE}\right)}^{\prime }{\left[Var\left({\hat{\beta }}_{FE}\right)-Var\left({\hat{\beta }}_{RE}\right)\right]}^{-1}\left({\hat{\beta }}_{FE}-{\hat{\beta }}_{RE}\right)$$ (6)

② Conduct hypothesis testing:

Null Hypothesis $H_{0}$ : ${\hat{\beta }}_{FE}={\hat{\beta }}_{RE}$ (This hypothesizes that the estimates are consistent, favoring the more efficient Random Effects model).

Alternative Hypothesis $H_{1}$ : ${\hat{\beta }}_{FE}\neq {\hat{\beta }}_{RE}$ (This hypothesizes that the estimates are inconsistent, favoring the consistent Fixed Effects model).

Under the null hypothesis and in large samples, the H statistic asymptotically follows a chi-squared distribution with a number of degrees of freedom that is equal to the number of parameters k being compared. If the calculated H value exceeds the critical chi-squared value (or if the corresponding *p*-value is less than the significance level), the null hypothesis is rejected. This indicates that the Fixed Effects model is more effective.

## 4 Results

### 4.1 Basic information

Descriptive statistics indicate a reduction in total healthcare expenditure between the pre- and post- policy implementation period (7.775 ± 6.468 to 6.075 ± 5.610). This is primarily attributable to a decrease in inpatient costs (3.825 ± 2.793 to 2.682 ± 2.470). However, outpatient expenses increase (1.583 ± 1.497 to 2.185 ± 2.019). Chronic disease-specific expenditures remain stable. The cohort shows a modest aging trend (mean age: 57.3–63.0 years), with consistent gender distribution, insurance type, and clinical grading over time (Table 3).

**Table 3:** Descriptive statistics.

Variable	Before	After
Expense_Total	7.775 ± 6.468	6.075 ± 5.61
Expense_OUT	1.583 ± 1.497	2.185 ± 2.019
Expense_CSD	1.215 ± 1.135	1.217 ± 1.123
Expense_INP	3.825 ± 2.793	2.682 ± 2.47
Age	57.341 ± 4.272	62.998 ± 4.152
Gender	0.452 ± 0.024	0.457 ± 0.031
Insutype	0.135 ± 0.122	0.129 ± 0.106
Grader	0.100	0.100
Level	0.200	0.200

### 4.2 Pooled ordinary least squares (OLS) regression results

To evaluate the impact of the global budget payment policy within the county’s integrated healthcare delivery system on medical insurance expenditures, baseline regressions were completed using OLS with cross-sectional data. To correct for potential within-group correlation, the regression results report both conventional standard errors and cluster-robust standard errors (Table 4). After controlling for relevant covariates, the policy implementation dummy variable is found to be significantly negatively correlated with total medical insurance fund expenditures (H1) and inpatient fund expenditures (H4) (*p* < 0.01). This result remains robust across different standard error specifications, supporting hypotheses H1 and H4. This indicates that the global budget policy has a restraining effect on both total expenditures and inpatient expenditures within this county-level integrated healthcare delivery system.

**Table 4:** Results of pooled OLS regression.

Variable	lnExpense_Total		lnExpense_OUT		lnExpense_CSD		lnExpense_INP	
	POLS	POLS_roust	Pooled OLS	POLS_roust	Pooled OLS	POLS_roust	Pooled OLS	POLS_roust
Post	−0.517^***^	−0.517^***^	0.145	0.145	−0.0991	−0.0991	−0.737^***^	−0.737^***^
	(−3.95)	(−4.38)	(1.08)	(1.09)	(−0.72)	(−0.71)	(−5.81)	(−7.02)
Age	0.0228^*^	0.0228^*^	0.0283^**^	0.0283^**^	0.0256^**^	0.0256^*^	0.0199^*^	0.0199^*^
	(1.89)	(1.80)	(2.28)	(2.07)	(2.01)	(1.84)	(1.70)	(1.68)
Gender	−6.624^***^	−6.624^***^	−6.886^***^	−6.886^***^	−6.956^***^	−6.956^***^	−6.247^***^	−6.247^***^
	(−3.98)	(−3.19)	(−4.03)	(−3.17)	(−3.95)	(−3.17)	(−3.87)	(−3.17)
Insutype	0.519	0.519	0.553	0.553	0.514	0.514	0.462	0.462
	(1.16)	(1.03)	(1.21)	(1.08)	(1.09)	(0.97)	(1.07)	(0.96)
GRADER	−1.701^***^	−1.701^***^	−1.731^***^	−1.731^***^	−1.763^***^	−1.763^***^	−1.659^***^	−1.659^***^
	(−10.23)	(−21.17)	(−10.14)	(−21.01)	(−10.03)	(−21.08)	(−10.29)	(−20.58)
_cons	6.517^***^	6.517^***^	4.659^***^	4.659^***^	4.583^***^	4.583^***^	5.860^***^	5.860^***^
	(5.46)	(4.69)	(3.80)	(3.18)	(3.63)	(3.09)	(5.06)	(4.47)
*N*	400	400	400	400	400	400	400	400
*R* ^2^	0.298	0.298	0.288	0.288	0.273	0.273	0.324	0.324
Adj. *R*^2^	0.289	0.289	0.279	0.279	0.264	0.264	0.315	0.315
*F*	33.38	202.4	31.94	230.1	29.56	216.8	37.69	177.5
*p*	2.11e−28	1.84e−106	2.56e−27	1.75e−114	1.71e−25	1.00e−110	1.42e−31	1.45e−98

*t*-statistics are reported in parentheses. ^*^p < 0.1, ^**^p < 0.05, ^***^p < 0.01.

The policy’s impact on outpatient expenditures (H2) and expenditures for chronic and special diseases (H3) is not statistically significant (*p* > 0.01). This indicates potential heterogeneity in policy effects across different types of services. In terms of model fit, the *R*^2^ values for the regressions range from 0.273 to 0.324, with adjusted *R*^2^ values between 0.264 and 0.315. This indicates that the models explain approximately 26–32% of the variation in expenditures. The *F*-tests are all significant at the *p* < 0.001 level. This supports the overall validity of the model specifications and the joint significance of the variables. This provides a reliable statistical foundation for subsequent analyses.

### 4.3 Fixed effects (FE) model regression results

To mitigate potential omitted variable bias due to time-invariant inherent heterogeneity across county-level integrated healthcare delivery systems, this study implemented fixed-effects models to more precisely identify the net effect of policy implementation (Table 5). After controlling for entity fixed effects, the core findings are further validated: policy implementation is significantly negatively associated with both total medical insurance expenditures (H1) and inpatient expenditures (H4) (*p* < 0.01). The results are robust across alternative standard error specifications. This supports the finding that the global budget reform effectively constrains aggregate and inpatient spending.

**Table 5:** Results of fixed effects regression.

Variable	lnExpense_Total		lnExpense_OUT		lnExpense_CSD		lnExpense_INP	
	FE	FE_roust	FE	FE_roust	FE	FE_roust	FE	FE_roust
Post	−0.429^***^	−0.429^***^	0.231^***^	0.231^**^	−0.0140	−0.0140	−0.653^***^	−0.653^***^
	(−14.490)	(−4.110)	(7.830)	(2.930)	(−0.570)	(−0.770)	(−16.460)	(−3.820)
Age	−0.003	−0.003	0.003	0.003	0.001	0.001	−0.05	−0.005
	(−0.87)	(−0.51)	(1.02)	(0.74)	(0.20)	(0.20)	(−1.18)	(−0.51)
Gender	0.646	0.646	0.506	0.506	0.504	0.504	0.875	0.875
	(1.590)	(0.940)	(1.250)	(0.760)	(1.500)	(0.910)	(1.610)	(1.040)
Insutype	0.350	0.350	0.393	0.393	0.591^***^	0.591	0.0702	0.0702
	(1.420)	(0.610)	(1.600)	(0.710)	(2.900)	(1.110)	(0.210)	(0.100)
Grader	0.000	0.000	0.000	0.000	0.000	0.000	0.000	0.000
	(0.000)	(0.000)	(0.000)	(0.000)	(0.000)	(0.000)	(0.000)	(0.000)
_cons	1.495^***^	1.495^***^	−0.491^**^	−0.491	−0.693^***^	−0.693^*^	0.969^***^	0.969
	(6.10)	(3.84)	(−2.02)	(−1.36)	(−3.43)	(−1.95)	(2.95)	(1.64)
*N*	400	400	400	400	400	400	400	400
*R* ^2^	0.474	0.474	0.231	0.231	0.031	0.031	0.538	0.538
Adj. *R*^2^	0.456	0.468	0.205	0.223	−0.002	0.021	0.522	0.533
*F*	86.88	11.69	28.92	9.539	3.091	2.013	112.2	6.315
*p*	1.42e−52	0.00130	4.84e−21	0.00269	0.0159	0.176	2.32e−63	0.0105

*t*-statistics are reported in parentheses. ^*^p < 0.1, ^**^p < 0.05, ^***^p < 0.01.

Under the fixed-effects specification, policy implementation is significantly positively associated with outpatient expenditures (H2) (*p* < 0.05). This indicates that after accounting for individual heterogeneity, the budget cap may lead to service restructuring, with potential cost-shifting from inpatient to outpatient care. For chronic/special disease expenditures (H3), policy implementation continues to show no statistically significant impact. This may indicate that these expenditures (driven by long-term treatment protocols and patient behavior) have a delayed response to budgetary constraints.

Regarding model fit, the total expenditure and inpatient expenditure models have strong explanatory power (*R*^2^ = 0.538 and 0.533, respectively). The chronic/special disease model shows a poor model fit (*R*^2^ = 0.031). This indicates that extraneous factors predominantly influence variations in the chronic/special disease model. Most models yield significant F-statistics (*p* < 0.01), confirming the joint significance of explanatory variables and overall model validity.

### 4.4 Least squares dummy variable (LSDV) model results

The study further applied the Least Squares Dummy Variable (LSDV) method for empirical analysis. Individual dummy variables control for time-invariant individual heterogeneity. This approach mitigates estimation bias caused by omitted variables or individual fixed effects, and increases the robustness of the model estimates. For the specific estimation, a dummy variable was generated for each hospital entity; the new variable was included in the regression model. Further, the regression analysis also reports results using cluster-robust standard errors to address potential problems of heteroskedasticity and autocorrelation and to assess the validity of the statistical inference. The estimates from the LSDV method support hypotheses H5-H6; they indicate the presence of significant individual effects and indicate that the fixed effects model is better than the pooled regression model (Table 6).

**Table 6:** Results of the least squares dummy variable model.

Variable	lnExpense_Total	lnExpense_OUT	lnExpense_CSD	lnExpense_INP
Post	−0.429^***^	0.231^***^	−0.0140	−0.653^***^
	(−15.55)	(7.69)	(−0.89)	(−14.64)
Age	−0.003	0.003	0.001	−0.005
	(−1.06)	(1.00)	(0.26)	(−1.31)
Gender	0.646	0.506	0.504	0.875
	(1.53)	(1.23)	(1.28)	(1.63)
Insutype	0.350	0.393	0.591^*^	0.0702
	(0.94)	(1.17)	(1.78)	(0.14)
ind_dum1	0.000	0.000	0.000	0.000
	(0.000)	(0.000)	(0.000)	(0.000)
ind_dum2	−3.041^***^	−3.111^***^	−3.193^***^	−2.937^***^
	(−59.43)	(−70.96)	(−82.11)	(−41.02)
ind_dum3	−0.408^***^	−0.404^***^	−0.448^***^	−0.398^***^
	(−8.93)	(−10.64)	(−12.74)	(−5.91)
ind_dum4	−0.808^***^	−0.729^***^	−0.778^***^	−0.783^***^
	(−23.99)	(−23.31)	(−35.13)	(−16.35)
ind_dum5	−1.032^***^	−1.109^***^	−1.051^***^	−1.043^***^
	(−33.45)	(−31.87)	(−42.06)	(−22.22)
ind_dum6	−0.983^***^	−1.016^***^	−1.037^***^	−0.945^***^
	(−13.60)	(−15.76)	(−17.53)	(−9.21)
ind_dum7	−2.053^***^	−2.111^***^	−2.090^***^	−2.044^***^
	(−43.05)	(−43.57)	(−48.80)	(−33.31)
ind_dum8	−1.805^***^	−1.801^***^	−1.799^***^	−1.795^***^
	(−45.58)	(−44.10)	(−57.40)	(−33.36)
ind_dum9	−3.126^***^	−3.173^***^	−3.325^***^	−2.944^***^
	(−36.14)	(−42.09)	(−52.83)	(−22.38)
ind_dum10	−3.050^***^	−3.121^***^	−3.209^***^	−2.939^***^
	(−62.74)	(−75.82)	(−90.30)	(−42.00)
_cons	3.126^***^	1.166^***^	0.999^***^	2.552^***^
	(11.05)	(3.96)	(3.84)	(7.16)
*N*	400	400	400	400
*R* ^2^	0.968	0.969	0.980	0.941
Adj. *R*^2^	0.967	0.968	0.979	0.939
*F*	996.8	1665.2	3187.1	479.9
*p*	1.28e−287	0	0	5.42e−229

*t*-statistics are reported in parentheses. ^*^p < 0.1, ^**^p < 0.05, ^***^p < 0.01.

### 4.5 Breusch–Pagan (B–P) test results

The Breusch–Pagan Lagrange Multiplier (LM) test is completed to help choose between the pooled OLS model and a variable-intercept model (i.e., a random or fixed effects model). The null hypothesis (H0) tested was that the variance of the individual effects for healthcare institutions within the CMAs is zero; the results indicate that the pooled OLS model is more appropriate. Table 7 shows that the test yields a *p*-value < 0.05, leading to the rejection of the null hypothesis that there are no individual effects at the 5% significance level. This confirms the presence of significant individual heterogeneity in the data. Therefore, a variable-intercept model (random or fixed effects models), which can capture such effects, is more effective than the pooled OLS model.

**Table 7:** Breusch–Pagan test results.

Category	Variance (Var)	Standard deviation (SD)	BP test *p*-value
Expense_Total (ln)	1.271	1.127	<0.01
Error term (e)	0.042	0.206	
Individual effect (u)	0.485	0.697	
Expense_OUT (ln)	1.325	1.151	<0.01
Error term (e)	0.042	0.204	
Individual effect (u)	0.537	0.733	
Expense_CSD (ln)	1.373	1.172	<0.01
Error term (e)	0.029	0.170	
Individual effect (u)	0.554	0.744	
Expense_INP (ln)	1.240	1.114	<0.01
Error term (e)	0.076	0.275	
Individual effect (u)	0.437	0.661	

### 4.6 Two-way fixed effects model results

There may be common time-based shocks during the study period (2022–2024), such as technological advancements, economic fluctuations, and adjustments in macro-level health policies. These may affect medical insurance fund expenditures of healthcare institutions. As such, this study further applies a two-way fixed effects model for empirical estimation. This means that the model incorporates time fixed effects in addition to individual fixed effects. This more accurately identifies the effect of the core explanatory variable, while simultaneously controlling for individual heterogeneity and time trends. Year-based dummy variables are introduced to absorb common temporal trends and external shocks across periods. This mitigates estimation bias arising from omitted time-invariant individual characteristics and time-evolving common factors, and increases the credibility and robustness of the hypothesis testing results. Table 8 presents the regression results from the two-way fixed effects model, and support the study hypotheses.

**Table 8:** Results of the two-way fixed effects model.

Variable	lnExpense_Total	lnExpense_OUT	lnExpense_CSD	lnExpense_INP
Post	−0.477^***^	0.186^**^	−0.0626^**^	−0.686^***^
	(−4.26)	(2.54)	(−3.19)	(−3.71)
Age	−0.004	0.003	−0.001	−0.006
	(−0.73)	(0.61)	(−0.12)	(−0.62)
Gender	0.404	0.227	0.240	0.610
	(0.74)	(0.44)	(0.58)	(0.86)
Insutype	0.380	0.420	0.623	0.0755
	(0.75)	(0.90)	(1.31)	(0.12)
1. nf	0.000	0.000	0.000	0.000
	(0.000)	(0.000)	(0.000)	(0.000)
2. nf	−0.0374^**^	−0.120^**^	−0.0882^***^	−0.0446
	(−2.77)	(−2.64)	(−3.57)	(−1.74)
3. nf	0.0036	−0.045	−0.023	−0.023
	(0.23)	(−1.01)	(−0.79)	(−0.95)
4. nf	0.102^**^	0.0500	0.0731^**^	0.0860
	(2.76)	(1.08)	(2.93)	(1.74)
_cons	1.690^***^	−0.271	−0.486^*^	1.197^*^
	(4.71)	(−0.95)	(−1.88)	(1.92)
*N*	400	400	400	400
*R* ^2^	0.506	0.307	0.148	0.554
Adj. *R*^2^	0.497	0.295	0.133	0.546
*F*	9.942	21.21	5.959	6.541
*p*	0.00132	0.0000656	0.00819	0.00597

*t*-statistics are reported in parentheses. ^*^p < 0.1, ^**^p < 0.05, ^***^p < 0.01.

### 4.7 Hausman test results

After determining the use of a variable-intercept model, the next step is to choose between the RE and FE models. The RE model requires that the explanatory variables be uncorrelated with the individual effects. In other words, it assumes strict exogeneity, which is a more stringent condition than the FE model. The FE model relaxes this assumption, but does so at the cost of losing more degrees of freedom. To choose the optimal model, this study applied the Hausman test for statistical inference. This test assesses whether there is a systematic difference between the estimated coefficients of the FE and RE models. The null hypothesis (H0) is that the RE model is correctly specified, meaning that the individual effects are uncorrelated with the explanatory variables. If the test results reject the null hypothesis, it indicates the FE model is more appropriate. The test constructs a chi-square statistic based on the differences in the estimated coefficients to statistically evaluate the model specification. The results indicate that the FE model is more suitable for the analysis, as detailed in Table 9.

**Table 9:** Results of the Hausman test.

Variable	Variable	(*b*)	(*B*)	(*b*–*B*)	Sqrt
		Fe 1	Re 1	Difference	Std. err.
Expense_Total (ln)	Post	−0.4291	−0.4293	0.0003	<0.001
	Age	−0.0026	−0.0025	−0.0001	0.003
	Gender	0.6458	0.6223	0.0235	<0.001
	INS_type	0.3496	0.3558	−0.0062	0.106
Expense_OUT (ln)	Post	0.2307	0.2304	0.0002	<0.001
	Age	0.0031	0.0032	−0.0001	0.002
	Gender	0.5057	0.4844	0.0213	<0.001
	INS_type	0.3928	0.3983	−0.0055	0.091
Expense_CSD (ln)	Post	−0.1402	−0.0142	0.0002	<0.001
	Age	0.0005	0.0006	−0.0001	0.002
	Gender	0.5038	0.4899	0.0139	<0.001
	INS_type	0.5908	0.5924	−0.0016	0.054
Expense_INP (ln)	Post	−0.6529	−0.6533	0.0004	<0.001
	Age	−0.0048	−0.0046	−0.0001	0.004
	Gender	0.8747	0.8273	0.0474	<0.001
	INS_type	0.0702	0.0888	−0.0185	0.144

## 5 Discussion

This study systematically evaluates the impact of the global budget payment policy for medical insurance funds within a county-level integrated healthcare delivery system on expenditures (12). The policy has played a positive role in controlling total medical insurance fund expenditures and optimizing the expenditure structure, although its effects are heterogeneous. First, the results from regression analysis (including models with ordinary and cluster-robust standard errors), FE models, the LSDV model, and the two-way fixed effects model consistently demonstrate that, following policy implementation, both the total medical insurance fund expenditures (H1) and inpatient fund expenditures (H4) within the medical alliances show statistically significant negative correlations (*p* < 0.01). This series of robust findings supports hypotheses H1 and H4, confirming that the global budget reform has a significant effect on medical insurance fund expenditures, particularly inpatient expenditures (13).

These outcomes align with the original intent of the policy: by introducing retained surpluses and shared responsibility for reasonable overruns, the external constraint on fund expenditures is internalized as an intrinsic managerial incentive within the medical alliances. This curbs unreasonable growth in medical costs, especially in inpatient services, which is a traditional area of high-cost growth (14). This indicates that, as a prepayment mechanism, the global budget can effectively guide healthcare institutions within the county-level medical alliance framework to shift from scale expansion to cost control and improved efficiency (15). Although the increase in outpatient costs may be related to enhanced primary care capacity and improved accessibility, in this study the cost growth is mainly concentrated in services transferred from inpatient to outpatient settings (such as intravenous infusion and rehabilitation therapy). During the same period, inpatient visits decreased while the average inpatient cost increased, suggesting more severe illness among hospitalized patients. This pattern is more consistent with ‘cost shifting’ than with simple growth. We also examined changes in primary outpatient volume and found no significant expansion in service volume, further ruling out the ‘capacity-driven growth’ explanation.

In contrast to the significant controlling effect on inpatient expenditures, there are different impact patterns on outpatient fund expenditures (H2) and chronic/special disease fund expenditures (H3). In the baseline regressions, neither are statistically significant. However, after controlling for individual fixed effects (in the FE model), outpatient fund expenditures (H2) show a significant positive correlation. This shift indicates that time-invariant individual characteristics of healthcare institutions may have masked or confounded the true policy effect on outpatient expenditures (16). After controlling for this individual heterogeneity, the policy may be associated with some growth in outpatient expenditures (17). This may be because, under the constraint of the global budget, medical alliances might shift some patients with mild conditions or in recovery phases (who might otherwise be hospitalized), to outpatient services. This service substitution may lead to increased outpatient expenditures (18). Payment reform may trigger complex adjustments in healthcare service delivery behaviors. Meanwhile, chronic/special disease fund expenditures (H3) are not statistically significant across any of the model specifications (19).

This study applies systematic model testing and comparisons to assess the robustness of its core conclusions. First, the Breusch–Pagan test rejects the pooled OLS model at the 5% significance level. This confirms significant individual heterogeneity in the data and justifies the use of models that capture individual effects (such as FE or RE models). Second, the Hausman test results indicate that the FE model is better than the RE model. This means that in this study, the individual effects of healthcare institutions (such as unobservable management capabilities and resource endowments) are likely to be correlated with the core explanatory variable (the global budget policy) (20). Using an RE model would lead to estimation bias. Therefore, the FE model more clearly identifies the policy effect.

Further, this study verifies the validity of the FE model specification using the LSDV method and introduces a two-way fixed effects model that simultaneously controls for time-invariant individual fixed effects and individual-invariant time fixed effects (such as annual macroeconomic conditions and nationwide health policy adjustments). The core results remain valid under the two-way fixed effects model; this significantly increases the credibility of the findings (21). This indicates that the decrease in medical insurance fund expenditures is more likely attributable to the implementation of the global budget policy than to unobserved individual characteristics or common time trends (22).

The study’s results point to important policy implications: (1) The global budget payment policy for medical insurance funds within county-level medical alliances effectively controls medical insurance fund expenditures, particularly the excessive growth of inpatient costs. The policy should be maintained and further promoted (23). (2) The policy effects are structural; while decreasing inpatient costs, there is a risk of services shifting to outpatient care. Medical insurance authorities should strengthen monitoring and incorporate outpatient expenditures into the overall budget assessment system to avoid a “seesaw effect” in cost control (24). (3) For special medical expenditures (e.g., chronic/special diseases), policymakers should explore composite payment methods that align with the global budget and emphasize health outcomes and long-term management (25). (4) When evaluating the effects of payment reform, econometric methods that effectively control for individual heterogeneity and time trends (such as the two-way fixed effects model) are needed to generate more reliable evidence for policy assessments and to provide a scientific basis for subsequent policy optimization (26).

Like all studies, this one has limitations (27). First, it has a relatively short observation period and may not fully capture the policy’s long-term dynamic effects. The data in this study spans 40 months, which is a medium-short-term observation. It cannot evaluate the long-term effects of the reform on medical expense structure, health outcomes, and patient behavior inertia. Longer time series data (such as over 5 years) are needed in the future to examine the sustainability and attenuation of the effects. Second, the study focuses on the total volume and structure of fund expenditures. Future research could further analyze the policy’s specific impacts on healthcare service quality, patient health outcomes, the revenue structure of healthcare institutions, and medical staff behaviors. Finally, regarding data, access to richer institutional-level characteristic variables (such as medical staff structure and equipment configuration) may provide deeper descriptions of the heterogeneous mechanisms through which the policy takes effect (28).

## 6 Conclusion

This study conducts a systematic econometric analysis to confirm that the implementation of the global budget payment policy for medical insurance funds within county-level integrated healthcare delivery systems has a significant and robust inhibitory effect on the total expenditure of medical insurance funds, particularly on inpatient costs. However, the policy effect is structurally heterogeneous. Outpatient expenditures may increase, highlighting the need to guard against the risk of cost shifting from inpatient to outpatient services. Expenditures for chronic and special diseases appear to be largely unaffected. The findings indicate that the policy effectively improves the efficiency of the healthcare service system. However, responses may differ across various service types. Refined monitoring and management measures are needed to balance the sustainability of medical insurance funds and the quality of healthcare services.

## Data Availability

The original contributions presented in the study are included in the article/supplementary material, further inquiries can be directed to the corresponding author.
